# Concomitant EGFR Mutations and ALK Rearrangements in Lung Adenocarcinoma Treated With Osimertinib

**DOI:** 10.7759/cureus.48122

**Published:** 2023-11-01

**Authors:** David Thomas, McKenzie E Maloney, Girindra Raval

**Affiliations:** 1 Hematology and Oncology, Medical College of Georgia, Augusta University, Augusta, USA

**Keywords:** treatment, osimertinib, alk mutation, egfr mutation, lung cancer

## Abstract

Lung cancer is the third most common cancer in addition to being the cancer responsible for the most annual deaths in the United States, comprising 15% of all diagnosed cancers, and 28% of all cancer deaths in 2020. Major advances in survival are because of gene sequencing and the advent of targeted biological therapy. The prevalence of epidermal growth factor receptor (EGFR) mutations coexisting with anaplastic lymphoma kinase (ALK) rearrangements is quite low. However, the clinical relevance and effective treatment of these cancers require further investigation. This case series describes two patients diagnosed with stage IV adenocarcinoma with coexisting EGFR and ALK rearrangements. In Case 1, a 73-year-old male presented with worsening ataxia and headaches. In Case 2, a 64-year-old female presented with worsening dyspnea. Molecular studies revealed ALK gene fusion and the L861Q EGFR mutation in Case 1 and L858R EGFR mutation and ALK gene fusion in Case 2. Both patients received a gamma knife and an EGFR-tyrosine kinase inhibitor (TKI), osimertinib. In one of the cases, following the discovery of new brain metastases, the dose of osimertinib was increased from 80 to 160 mg. The patient passed away nine months after beginning EGFR-TKI treatment, one month after increasing the dose. The second patient experienced a significant interval reduction in the size of enhancing metastasis in both the right frontal and left parietal lobe after four months of EGFR-TKI treatment. The cases of coexisting EGFR mutations and ALK rearrangements are quite rare, and treatment can be challenging. Here, EGFR-TKI had a mixed response among our patients.

## Introduction

Lung cancer has the highest mortality of all cancers in the world. According to the SEER database, lung cancer accounted for 130,180 deaths in 2022, which is more than double colorectal cancer, which is the next most common cause of death for individuals from cancer [1]. Recent advances in identifying oncogenic driver mutations have revolutionized cancer treatment and led to the development of targeted therapies for non-small cell lung cancer (NSCLC) [2]. While the prevalence varies among different populations, it has been shown that the epidermal growth factor receptor (EGFR) gene mutation is one of the most frequent drivers, as it is present in around 15% of cases of NSCLC in African Americans and Caucasians and up to 40% of cases in those of Asian descent [3]. The most common mutations in the EGFR gene are the exon 19 deletions, which account for around 44% of all EGFR lung cancers, and the exon 21 L858R substitution, which is responsible for another 47% of EGFR cases [4]. Atypical mutations include point mutations such as L861Q and exon 20 insertions [5,6]. Mutations in the EGFR tyrosine kinase have become the target for many new drugs. Additionally, rearrangements in the ALK (anaplastic lymphoma kinase) gene have been identified in 5-6% of NSCLC [7]. There is a rare subtype of NSCLC patients that have both EGFR mutations and ALK rearrangements [8,9].

The treatment for both driver mutations' associated lung cancers is different. Based on recent guidelines, metastatic lung cancer patients with EGFR mutations, specifically both the exon 19 EGFR deletion and the EGFR L858R substitution driver mutation, are best treated with upfront osimertinib based on the FLAURA Trial [10]. First-line treatment for metastatic lung cancer patients with ALK gene rearrangement, specifically the EML4-ALK gene fusion on the short arm of chromosome 2, comprises alectinib and ceritinib [11,12], with crizotinib being another option, based on the PROFILE study [13]. Here, we report two cases of concomitant EGFR mutation and ALK rearrangements and their treatment in patients with multifocal NSCLC with brain metastasis.

## Case presentation

Case 1

**Figure 1:**
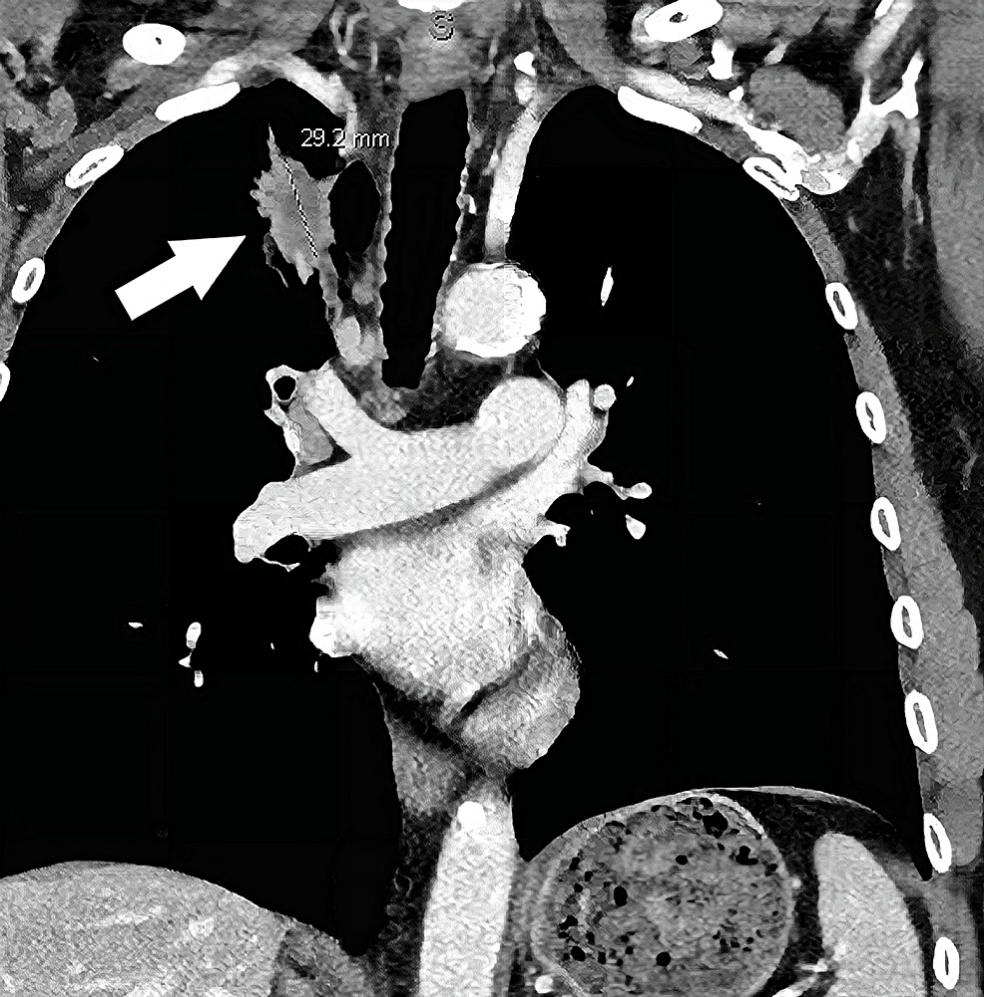
CT Chest at the Time of Diagnosis Shown here is the original imaging revealing a right upper lobe spiculated pulmonary lesion measuring 29.2 mm in diameter. This imaging was obtained after concerns for a primary lung tumor arose and was utilized in diagnosing the patient as the imaging confirmed the suspicion.

A 73-year-old male with a past medical history of smoking, hypertension, prostate cancer, hyperlipidemia, and coronary artery disease presented to the clinic for worsening headaches and ataxia. A workup revealed that he had three cerebellar masses. CT chest/abdomen and pelvis showed a right upper lobe spiculated pulmonary lesion suspicious of primary lung malignancy (Figure 1). FISH study of lung biopsy showed ALK gene rearrangement in 30% of cells. Additionally, the L681Q EGFR mutation was detected.

**Figure 2:**
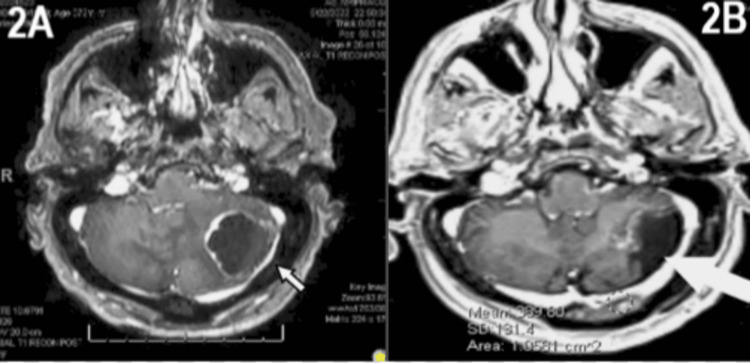
Brain Imaging Showing Cerebellar Metastases at Different Stages of Treatment 2A: Original MRI of the brain taken immediately following diagnosis, which revealed cerebellar metastasis. This image was taken prior to initiating treatment to monitor for possible brain metastases, and, upon review, it was concluded that the malignancy had metastasized to the cerebellum at the time of presentation. 2B: Follow-up MRI was done after the patient received treatment with both SRS (stereotactic radiation surgery) for the brain metastases and eight months of osimertinib to treat the patient’s lung tumor and metastases. This imaging showed growth and further depth of invasion of the original cerebellar mass observed eight months prior to the time of diagnosis.

**Figure 3:**
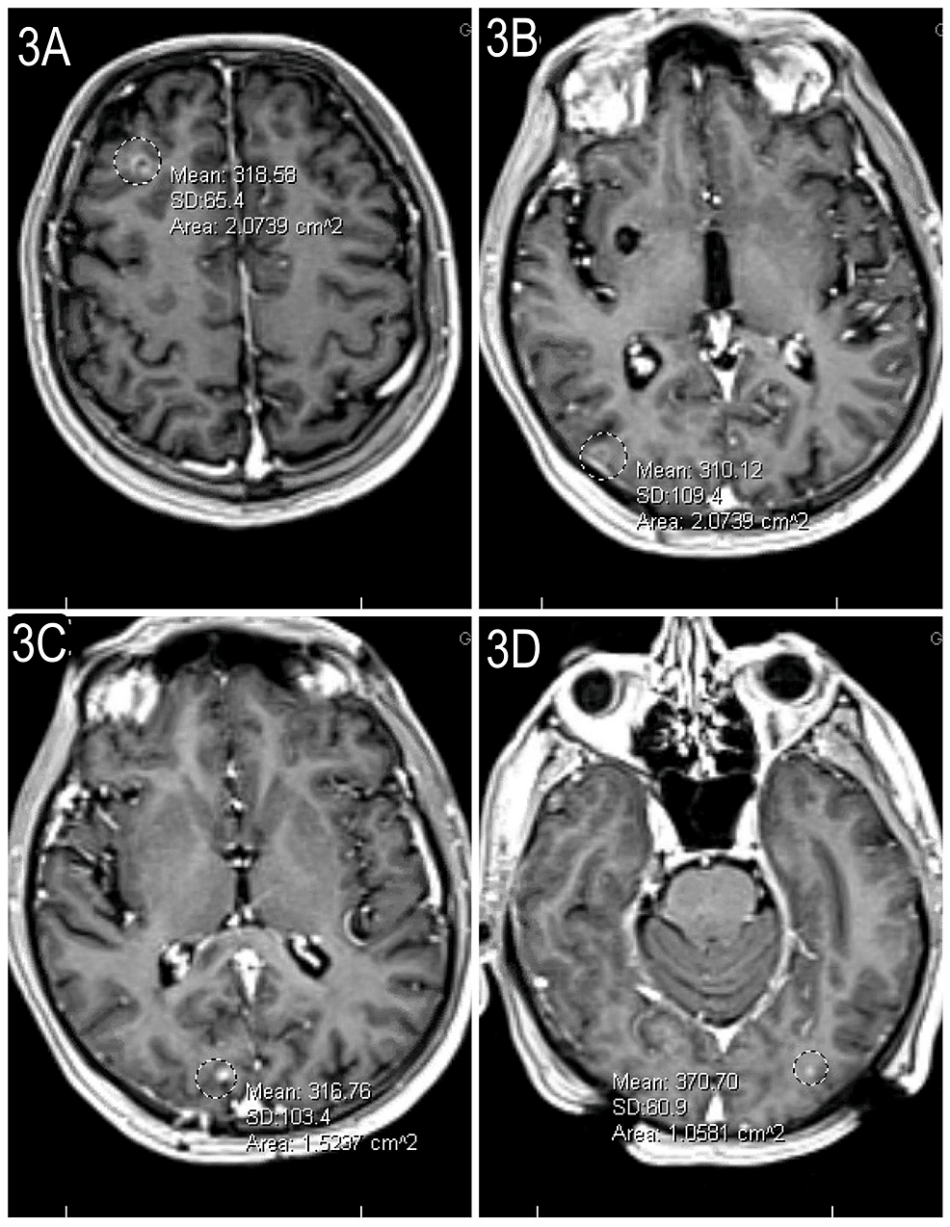
Newly Identified Brain Masses Discovered Following Eight Months of Treatment Signifying Disease Progression 3A-3D: These images were collected from the brain MRI the patient received following eight months of osimertinib to monitor the progression of the original brain metastasis in addition to checking for potential new masses. These images show different new brain metastases that were discovered throughout the brain eight months after initial diagnosis and discovery of spread to the brain. Following eight months of treatment, multiple new masses were identified, which are visualized in this figure, signifying the progression and further spread of the disease despite treatment.

**Figure 4:**
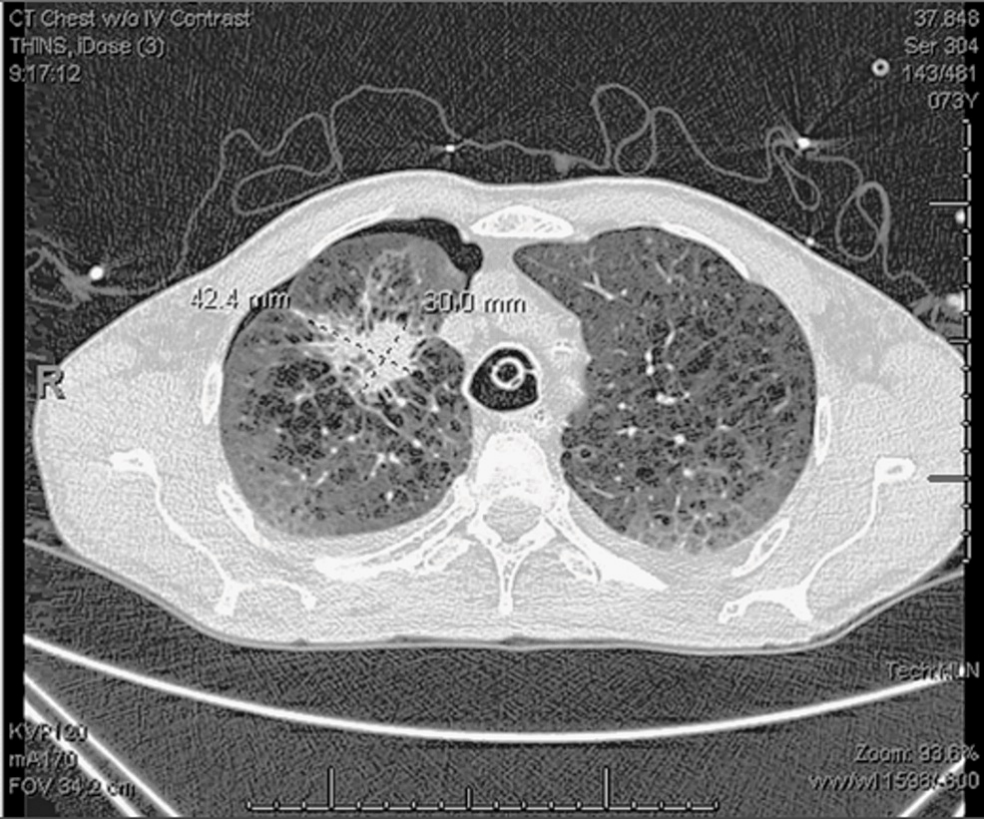
CT Scan of the Chest Following Eight Months of Treatment with Osimertinib This image was taken from a CT scan following eight months of treatment with osimertinib. The imaging study was conducted to monitor the progression of the disease during follow-up for the patient's lung tumor diagnosed eight months ago. The mass shown here corresponds to the original right upper lung mass detected on diagnostic imaging eight months ago. This imaging confirmed the growth of the original lesion and the progression of the disease, indicating that the malignancy was not responding to treatment and continuing to progress. Following this scan, osimertinib was increased from 80 mg to 160 mg to stop the progression of the disease.

The patient underwent suboccipital craniotomy for the resection of cerebellar lesions (Figure 2A) that were positive for Napsin and CK7. He was diagnosed with stage IV T1cN0M1c lung adenocarcinoma positive for the L861Q EGFR driver mutation with multiple brain metastases. Osimertinib, an EGFR-tyrosine kinase inhibitor (EGFR- TKI), was started [10]. The patient underwent SRS (stereotactic radiation surgery) for the remaining brain lesions. At subsequent follow-ups, he tolerated the drug well and denied any side effects. A follow-up MRI eight months following the initiation of osimertinib showed disease progression with progression in size of the original cerebellar metastasis (Figure 2B). Additionally, disease progression was shown by the presence of new brain metastases on this MRI (Figures 3A, 3D). Additionally, the increasing size of a known lung mass was seen on a follow-up CT scan at eight months also concerning the disease progression (Figure 4). The dose was increased from 80 to 160 mg of osimertinib. The patient passed away nine months after beginning osimertinib treatment and one month after increasing the dose.

Case 2

**Figure 5:**
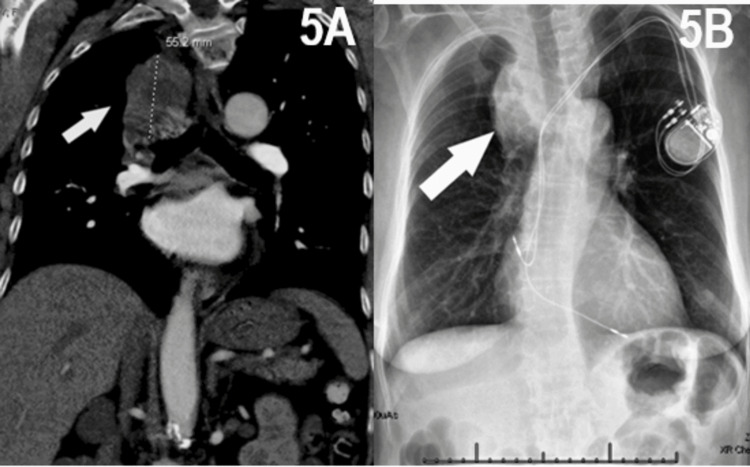
Chest CT (Figure 5A) Taken at the Time of Diagnosis and Chest X-ray (Figure 5B) Taken Two Months After the Initial Diagnosis, Both Demonstrating the Patient's Primary Right Upper Lung Mass 5A: Chest CT taken at the time of diagnosis prior to initiating the treatment identified a right upper lobe mass measuring 5.4*5.1 cm with central areas of necrosis, showing an interface with the trachea and esophagus and encasing the right upper lobe pulmonary artery with extension into the right main bronchus. This confirmed the suspicion of primary lung mass and was the initial diagnostic imaging. Follow-up imaging was conducted two months later. 5B: Chest X-ray taken two months after the initial identification of a right upper lung mass concerning malignancy. This imaging further progression of the previously identified mass with metastasis to paratracheal and 11 L and 4L thoracic lymph nodes, confirming stage IVC adenocarcinoma in this patient.

**Figure 6:**
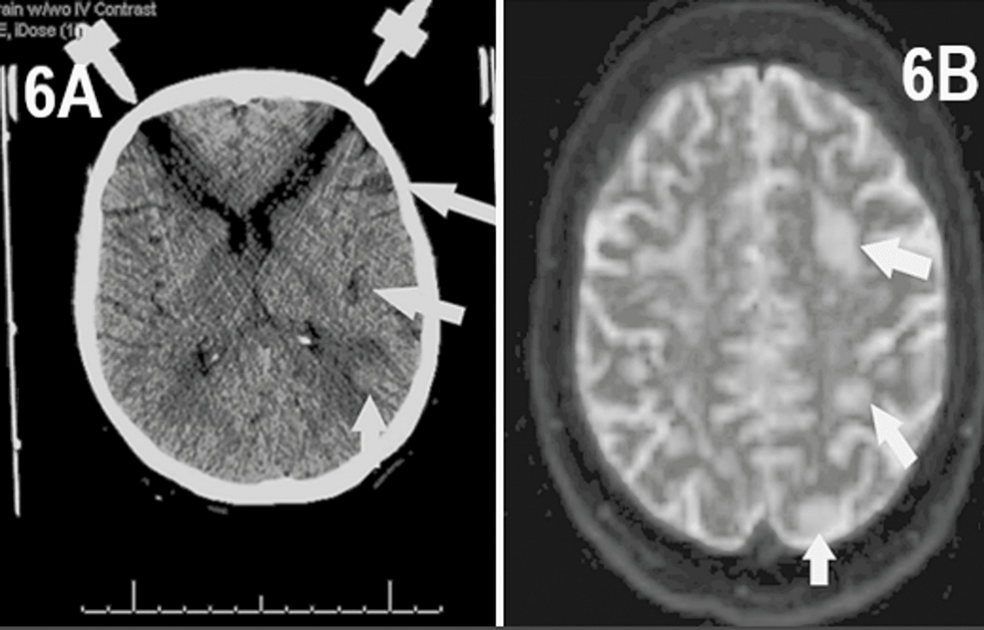
CT Head Conducted One Month After the Identification of Pulmonary Mass (Figure 6A) and MRI Brain Conducted Four Months After Identification (Figure 6B), Both Images Showing the Presence and Progression of Brain Metastases 6A: CT Head conducted one month after the identification of a pulmonary mass. This imaging was conducted to find potential brain metastases after the identification of a pulmonary mass concerning for lung cancer. The imaging identified a hypodense left inferior parietal lobe lesion measuring 1.5*1.1*1.4 cm along with a right inferior enhancing frontal gyrus lesion measuring 1.0*0.9*1.1 cm and an enhancing left superior parietal lobule lesion measuring 1.2*1.0*1.1 cm, confirming the presence of brain metastases and disease progression. 6B: MRI brain conducted four months after the identification of pulmonary mass and three months after the identification of brain metastases. This imaging was conducted to monitor the progression of brain metastases after continued treatment with both SRS and osimertinib. An MRI showed a significant reduction in the size of enhancing metastasis in both the right frontal and left parietal lobe, confirming positive response and improvement after receiving treatment.

A 64-year-old female with a past medical history of congestive heart failure, anemia, cerebral vascular accident, dyslipidemia, hypertension, degenerative joint disease, and chronic obstructive pulmonary disease with a 70 pack-year history presented to the emergency department (ED) following a fall with complaints of back pain over the thoracic and lumbar spine. A CT Spine and CT chest with contrast noted a right upper lobe mass measuring 5.4*5.1 cm with central areas of necrosis, showing interface with the trachea and esophagus and encasing the right upper lobe pulmonary artery with extension into the right main bronchus (Figure 5A). The patient was then managed in the surgical oncology clinic where she endorsed worsening shortness of breath during the preceding months, several episodes of hemoptysis, and a 30-pound unintentional weight loss over the prior three months. A follow-up CT head a month after presentation showed findings that are concerning for brain metastasis with a hypodense left inferior parietal lobe lesion measuring 1.5*1.1*1.4 cm along with a right inferior enhancing frontal gyrus lesion measuring 1.0*0.9*1.1 cm and an enhancing left superior parietal lobule lesion measuring 1.2*1.0*1.1 cm (Figure 6A). Additionally, a PET scan showed a 7.2* 5.8*9 cm necrotic hypermetabolic right upper lobe/right perihilar mass in addition to mild-to-moderate hypermetabolic perivascular and right hilar lymph nodes concerning regional metastatic lymphadenopathy.

Two months after presentation, the patient received rigid bronchoscopy and endobronchial ultrasound bronchoscopy for tumor debulking and pathologic diagnosis, which showed pathology consistent with metastatic pulmonary adenocarcinoma. Additionally, imaging showed metastasis to paratracheal and 11L and 4L thoracic lymph nodes confirming stage IVC adenocarcinoma in this patient (Figure 5B). Using FISH, 20% of the patient’s tumor cells were positive for ALK gene rearrangement. Additionally, an L858R EGFR mutation was detected on molecular testing. 

The patient received SRS for brain metastases and was started on osimertinib for the treatment of her lung cancer. She experienced severe diarrhea up to 6 episodes daily, lightheadedness, dizziness, and falls. Therefore, her treatment was held for two weeks, and the dose was reduced from 80 to 40 mg, which improved her symptoms. 

At a four-month follow-up after starting the EGFR-TKI, a brain MRI showed a significant interval reduction in the size of enhancing metastasis in both the right frontal and left parietal lobe (Figure 6B).

## Discussion

The EGFR mutation and ALK rearrangement are two of the more common driver mutations in NSCLC with an EGFR representing 15-40% of cases and ALK being present in approximately 5% of cases [3,14]. EGFR is a tyrosine kinase (TK) gene that can be transactivated by G protein-coupled receptors whose mutation leads to uncontrolled cell growth and proliferation. There are several known EGFR mutations that initiate tumor growth; these include EGFR exon 19 deletion in about 44% of all EGFR lung cancer and exon 21 L858R substitution in 47% and other less common variants [4]. Targeted therapies, EGFR-TKIs (epidermal growth factor receptor tyrosine kinase inhibitor), inhibit the overactivated tyrosine kinase genes, inhibiting uncontrolled cell growth.

First-generation EGFR TKIs include erlotinib, gefitinib, and icotinib, which bind reversibly, blocking downstream effects of TK. Afatinib and dacomitinib are second-generation and bind irreversibly via homodimers and heterodimers to the epidermal growth factor family of receptor tyrosine kinases (ErbB) family of receptors. Osimertinib, rociletinib, olmutinib, and lazertinib are third-generation. Finally, the third generation of TKIs specifically targets T790M EGFR mutant tumors, which is especially important as this is the most common mechanism of resistance occurring in half of all patients receiving first- or second-generation TKIs [15].

The AURA3 trial compared third-generation TKI, osimertinib, to pemetrexed with a platinum-based chemotherapy agent. Of 419 patients with T970M positive advanced NSCLC with disease progression on first-line EGFR-TKI, osimertinib significantly increased progression-free survival by 70% (hazard ratio; 0.30; 95% confidence interval (CI), 0.23 to 0.41; P<0.001). Further, among the 144 patients with brain metastases, osimertinib significantly increased progression-free survival by 70% [16]. Additionally, the FLAURA trial compared initiating treatment with osimertinib to other EGFR TKIs in patients with untreated advanced NSCLC. In this trial of 556 patients with the EGFR L858R exon 19 deletion, osimertinib increased overall survival by a median of 38.6 months compared to 31.8 months with erlotinib or gefitinib. Serious adverse events of grade 3 or higher were overall lower in the osimertinib group at 42% compared to the first-generation TKIs at 47% (hazard ratio, 0.80; 95.05% CI, 0.64 to 1.00; P=0.046) [17].

ALK rearrangement is a driver mutation in 6% of NSCLC, and uniquely, ALK rearrangements are more common in younger patients with the median age at diagnosis being 52 compared to a median age of 71 for lung cancer overall. ALK rearrangement is also more common in patients with little to no smoking history [13]. ALK is a tyrosine kinase gene located on the short arm of chromosome 2 which activates many downstream signaling pathways resulting in increased cell proliferation and survival. The ALK rearrangement results in a fusion of EML4-ALK, which can be a chemotherapeutic target. As fusion can occur in several different ways, there are many EML4-ALK variants that can drive disease. Targeted therapies against ALK TK rearrangements include crizotinib and alectinib. While therapeutics targeting this EML4-ALK rearrangement have proven beneficial, its detection has proven to be challenging owing to many variants [18].

Techniques such as fluorescent in-situ hybridization, immunohistochemistry, real-time polymerase chain reaction, and next-generation sequencing have all been used for detection, but they have their own challenges. Recently, a new technique has become more popular with the addition of plasma-based cfDNA techniques, also known as “liquid biopsy,” which offers a way to get a minimally invasive and specific diagnosis and has become an effective novel method to detect these ALK rearrangements. Among lung cancer patients with EGFR and ALK mutations and rearrangements, the sensitivity and specificity were 98.5% and 98.9%, respectively [19]. At this time, a positive liquid biopsy can confirm ALK rearrangement, and it is sufficient to begin ALK-targeted therapy [19].

Until recently, EGFR mutations and ALK rearrangements in lung cancer were thought to occur exclusively. The incidence of coexisting EGFR and ALK mutations is estimated to be 0.06%-1.6% of NSCLC cases, most frequently occurring in both unifocal and multifocal adenocarcinoma [18]. With advancements in detection, though, the incidence of coexisting EGFR mutations and ALK rearrangements is hypothesized to increase. A few cases have been identified with coexisting mutations. Patients with coexisting mutations have worsened prognoses when compared to either mutation alone [18,20]. A comprehensive review of over one hundred cases with co-occurring mutations also showed epidemiology such as that of EGFR mutation alone including a high prevalence of both women and never smokers being impacted by both mutations [20]. This review also noted that getting a true understanding of the prevalence of co-occurrence, and its clinical impact is challenging right now because of limited information and conflicting reports from studies that do exist on prevalence [20]. Case reports have described treatment in patients with NSCLC with both mutations. This co-occurring phenomenon can complicate diagnosis and treatment because many novel therapies focus on targeting one, not both, of the mutations, which may contribute to their poor prognosis. Some cases have described the initial use of traditional chemotherapeutics, such as folate antagonists and platin-based drugs, followed by targeted therapies upon disease metastasis [20]. However, most reported cases utilize an EGFR-TKI first line and employ ALK-TKI as a second line or beyond therapy. This treatment regime has a median progression-free survival of 5.8 months, but it was limited by a sample size of four. However, more research is needed to compare the use of individual EGFR and ALK inhibitors to discern differences in treatment efficacy.

## Conclusions

Because of the rare occurrence and novel discovery of coexisting EGFR mutations and ALK rearrangements, conclusive evidence regarding therapy is not readily available. In our cases, the patients were both diagnosed with Stage IV adenocarcinoma with coexisting EGFR and ALK mutations, and they were treated with osimertinib. The patient with the L861Q EGFR mutation tolerated treatment well, but the disease continued progressing within the four-month follow-up period. Our patient with the L858R EGFR mutation experienced severe side effects from the medication but showed regression of several metastases at the two-month follow-up. The mixed response to EGFR-TKI therapy our patients show highlights the need for further reports and evaluation of treatments for concomitant EGFR and ALK mutations in NSCLC.

## References

[1] (2022). Cancer facts & figures 2022. https://www.cancer.org/research/cancer-facts-statistics/all-cancer-facts-figures/cancer-facts-figures-2022.html.

[2] Sequist LV, Bell DW, Lynch TJ, Haber DA (2007). Molecular predictors of response to epidermal growth factor receptor antagonists in non-small-cell lung cancer. J Clin Oncol.

[3] Arcila ME, Nafa K, Chaft JE (2013). EGFR exon 20 insertion mutations in lung adenocarcinomas: prevalence, molecular heterogeneity, and clinicopathologic characteristics. Mol Cancer Ther.

[4] Graham RP, Treece AL, Lindeman NI, Vasalos P, Shan M, Jennings LJ, Rimm DL (2018). Worldwide frequency of commonly detected EGFR mutations. Arch Pathol Lab Med.

[5] Massarelli E, Johnson FM, Erickson HS, Wistuba II, Papadimitrakopoulou V (2013). Uncommon epidermal growth factor receptor mutations in non-small cell lung cancer and their mechanisms of EGFR tyrosine kinase inhibitors sensitivity and resistance. Lung Cancer.

[6] Devarakonda S, Morgensztern D, Govindan R (2015). Genomic alterations in lung adenocarcinoma. Lancet Oncol.

[7] Fan J, Wu J, Huang B, Zhu Y, Shi H, Dai X, Nie X (2020). Concomitant EGFR mutation and ALK rearrangement in multifocal lung adenocarcinoma: a case report. Diagn Pathol.

[8] Yin Q, Guo T, Zhou Y, Sun L, Meng M, Ma L, Wang X (2022). Effectiveness of alectinib and osimertinib in a brain metastasized lung adenocarcinoma patient with concurrent EGFR mutations and DCTN1-ALK fusion. Thorac Cancer.

[9] Soria JC, Ohe Y, Vansteenkiste J (2018). Osimertinib in untreated EGFR-mutated advanced non-small-cell lung cancer. N Engl J Med.

[10] Peters S, Camidge DR, Shaw AT (2017). Alectinib versus crizotinib in untreated ALK-positive non-small-cell lung cancer.. N Engl J Med.

[11] Soria JC, Tan DSW, Chiari R (2017). First-line ceritinib versus platinum-based chemotherapy in advanced ALK-rearranged non-small-cell lung cancer (ASCEND-4): a randomised, open-label, phase 3 study. Lancet (London, England).

[12] Solomon BJ, Mok T, Kim DW (2014). First-line crizotinib versus chemotherapy in ALK-positive lung cancer. N Engl J Med.

[13] Chia PL, Mitchell P, Dobrovic A, John T (2014). Prevalence and natural history of ALK positive non-small-cell lung cancer and the clinical impact of targeted therapy with ALK inhibitors. Clin Epidemiol.

[14] Caponnetto S, Cantale O, Friedlaender A (2021). A comparison between first-, second- and third-generation epidermal growth factor receptor tyrosine kinase inhibitors in patients with non-small-cell lung cancer and brain metastases. J Mol Pathol.

[15] Cataldo VD, Gibbons DL, Pérez-Soler R, Quintás-Cardama A (2011). Treatment of non-small-cell lung cancer with erlotinib or gefitinib. N Engl J Med.

[16] Mok TS, Wu Y-L, Ahn M-J (2017). Osimertinib or platinum-pemetrexed in EGFR T790M-positive lung cancer. N Engl J Med.

[17] Shaw AT, Kim DW, Mehra R (2014). Ceritinib in ALK-rearranged non-small-cell lung cancer. N Engl J Med.

[18] Cipriano É, Magalhães H, Tavares C, Pinto J, Cirnes L, Estevinho F (2021). Concurrent EGFR mutation and ALK rearrangement in stage IV lung adenocarcinoma—a case report and a literature review. Porto Biomed J.

[19] Garcia J, Kamps-Hughes N, Geiguer F, Couraud S, Sarver B, Payen L, Ionescu-Zanetti C (2021). Sensitivity, specificity, and accuracy of a liquid biopsy approach utilizing molecular amplification pools. Sci Rep.

[20] Lo Russo G, Imbimbo M, Corrao G (2017). Concomitant EML4-ALK rearrangement and EGFR mutation in non-small cell lung cancer patients: a literature review of 100 cases. Oncotarget.

